# Role of genetic variations of *chitinase 3*-*like 1* in bronchial asthmatic patients

**DOI:** 10.1186/s12948-018-0086-7

**Published:** 2018-04-02

**Authors:** Kazuyuki Abe, Yutaka Nakamura, Kohei Yamauchi, Makoto Maemondo

**Affiliations:** 1 Division of Pulmonary Medicine, Allergy, and Rheumatology, Department of Internal Medicine, Iwate Medical University School of Medicine, 19-1 Uchimaru, Morioka, 0208505 Japan; 2 Department of Allergy and Rheumatology, Nippon Medical School Graduate School of Medicine, 1-1-5 Sendagi, Tokyo, 1138603 Japan

**Keywords:** Bronchial asthma, Single nucleotide polymorphisms, Chitinase 3-like 1

## Abstract

**Background:**

Single nucleotide polymorphisms (SNPs) in *chitinase 3*-*like 1* (*CHI3L1*) are associated with bronchial severity and pulmonary function. CHI3L1 proteins are involved in both innate and adaptive immune responses; however, to date, the correlation of these SNPs and their age of onset of bronchial asthma has not been demonstrated.

**Methods:**

To address the role of these genetic variations, 390 patients with well-controlled bronchial asthma and living in Japan were recruited, genotyped, and had a pulmonary function test performed on them in this study. To analyze the concentration levels of CHI3L1 protein, bronchial lavage fluids were examined.

**Results:**

Forced expiratory volume in one second, %predicted (%FEV1), was significantly decreased in homozygotes of rs1214194 compared to heterozygotes and wild type. The age of onset of adult bronchial asthma was significantly younger in GG homozygotes of rs4950928 and AA homozygotes of rs1214194 than in the other two genotypes. The concentration of CHI3L1 protein in bronchial lavage fluid increased in both homozygotes of rs4950928 and rs1214194.

**Conclusions:**

Our study demonstrated that the homozygotes of rs4950928 and rs1214194 of *CHI3L1* might predict an early onset of bronchial asthma and have the propensity to promote airway remodeling.

*Trial registration* JMA-IIA00045 remodeling-ICS

## Background

Bronchial asthma is a disorder of the conducting airways that leads to variable airflow obstructions in association with airway hyperresponsiveness and a local accumulation of inflammatory cells, particularly Th2-type lymphocytes, eosinophils, and mast cells [1]. Allergens, such as those from mite and fungal exposure, up-regulate adaptive and innate immune responses, leading to the production of proinflammatory and profibrotic factors that may ultimately contribute to airway remodeling [2, 3]. Polysaccharide chitin, which is a polymer of *N*-acetylglucosamine, is found in the walls of fungi; exoskeleton of crabs, shrimp, and insects; the microfilarial sheath of parasitic nematodes; and the lining of the digestive tracts of many insects [4–8]. Chitinases are the enzymes that digest chitin polymer, and human subjects have 2 chitinases encoded in their genome: chitotriosidase and acidic mammalian chitinase (AMCase). AMCase and the chitinase-like protein YKL-40/chitinase 3-like 1 (CHI3L1), which lacks chitinase activity, were shown to play a critical role in inflammation driven by Th2-type cells and were expressed at high levels in tissues from patients with asthma [9–11]. YKL-40/CHI3L1 are produced by a variety of cells, including neutrophils, monocytes, macrophages, chondrocytes, synovial cells, endothelial cells, and tumor cells [12, 13]. Serum [10], lung, bronchial tissues [14], and sputum [15] have been noted in patients with bronchial asthma. Moreover, single nucleotide polymorphisms (SNPs) in *CHI3L1* have been associated with a risk of bronchial asthma, bronchial asthma severity, and pulmonary function in populations of European ancestry [15]. Although it appears that YKL-40/CHI3L1 is strongly associated with both innate and adaptive immune responses [16], the correlation of these SNPs and the age of onset of bronchial asthma has not been demonstrated. In the current study, we aimed to assess whether variants in the *CHI3L1* rs4950928 and rs1214194 genotypes were associated with lung function, the age of onset, and the airway expression of CHI3L1 protein in Japanese adult asthmatic patients.

## Methods

### Study subjects

All study subjects were recruited from the Iwate Medical University Hospital. Patients aged ≥ 18 years were eligible if they had a diagnosis of asthma as defined by the American Thoracic Society criteria for ≥ 5 years and were using inhaled corticosteroid (ICS) at a stable dose for ≥ 1 year before screening. Well-controlled asthmatic patients who had no other medical disorders, who had smoked less than 10-pack-years, and who had not been exposed to environmental hazards were considered for the study to exclude concomitant COPD. Well-controlled asthma symptoms were defined as meeting none of the following criteria in the previous 4 weeks [17]: (1) daytime asthma symptoms showing more than twice/week, (2) any night walking due to asthma, (3) a reliever needed for symptoms more than twice/week, and (4) any activity limitation due to asthma. This study was approved by the Iwate Medical University Hospital Ethics Committee (H20-119) and registered with Clinical Trials (JMA-IIA00045 remodeling-ICS). Prospective patients were notified of our desire to include them in our study and were asked if they would be willing to participate. Upon acceptance, the subjects provided written informed consent according to the ethical protocols of our institution. Subjects were assessed for age, height, body weight, sex, age of onset, eosinophil counts, serum IgE concentration, and spirometry. The data for age of onset of bronchial asthma were self-reported. Spirometry was performed (HI-801, CHEST, Tokyo, Japan) according to the ERS/ATS Guidelines [18]. Airway methacholine responsiveness was measured using an Astograph (Jupiter 21, CHEST, Tokyo, Japan) according to the method described by Takishima et al. [19]. The examination was performed by measuring dose–response curves of respiratory resistance during continuous inhalation of methacholine at a stepwise incremental concentration. Methacholine hydrochloride in isotonic saline was gradually increased to 49, 98, 195, 390, 781, 1563, 3125, 6250, 12,500, and 25,000 μg/mL [20]. DNA was isolated from lymphocytes using standard procedures. Subjects were genotyped for rs4950928 and rs1214194 using a 7500 Fast Real-Time PCR System (Life Technologies Japan, Tokyo, Japan).

### Fiberoptic bronchoscopy and specimen handling

Asthmatic patients underwent fiberoptic bronchoscopic examinations during ICS treatment. Asthmatic bronchial lavage fluids (BLF) were obtained from (i) patients harboring CC (wild type) of rs4950928; (ii) patients harboring GG (homozygous) of rs4950928; (iii) patients harboring GG (wild type) of rs1214194; and (iv) patients harboring AA (homozygous) of rs1214194. Bronchial lavage was performed by inserting a flexible fiberoptic bronchoscope (Olympus; Olympus Optical Co Ltd, Tokyo, Japan) under local anesthesia, as previously described [21]. BLF was extracted from one of the subsegmental bronchi of the left lingular division by injection of 20 mL aliquots of sterile saline pre-warmed to 36.5 °C twice and gently aspirated back into polypropylene tubes kept on ice. We obtained 20–25 mL of BLF from each asthma patient. Immediately after lavage, mucus was removed from the fluid by filtration through gauze, total and differential cell counts were performed, and the fluid was then centrifuged at 200×*g* for 10 min at 4 °C. The supernatant was decanted and stored at − 80 °C. Ten milliliters of BLF supernatant was concentrated to 1.0 mL (tenfold) by centrifugation using Centrifugal Filter Devices (Amicon Ultra-0.5, Merck Millipore, Darmstadt, Germany). CHI3L1 levels were measured in duplicate in BLF specimens using a commercially available ELISA kit for Human Chitinase 3-like 1 Immunoassay (R&D Systems, Inc., Minneapolis, MN, USA). The mean value of the 2 duplicates was used in the statistical analyses. Duplicate samples with coefficients of variation greater than 20% were reassayed.

### Statistical analysis

Statistical analyses were performed using JMP version 11 (SAS Institute Inc., Tokyo, Japan). All data were expressed as the mean ± standard error. Comparisons of the patients’ characteristics between the three groups were performed using one-way ANOVA. Post hoc multiple comparisons were performed using the Tukey–Kramer test for differences among all groups. Comparisons of the total and differential cell counts, and the concentration of CHI3L1 in BLF were performed using a *t*-test. *P* values < 0.05 were considered significant.

## Results

### Subject demographics and enrolment

Of 390 asthmatic patients screened, 381 of rs4950928 and 368 of rs1214194 were successfully genotyped (≥ 94%). We identified 270, 90, and 21 subjects that had CC wild type, CG heterozygotes, and GG homozygotes, respectively, for rs4950928 (Table 1). There were no significant differences in age at study enrolment, sex, eosinophil count status, serum IgE concentration, pulmonary function, or receiving dose of ICS. We also identified 170, 157, and 41 subjects that had GG wild type, GA heterozygotes, and AA homozygotes, respectively, for rs1214194 (Table 2). AA homozygotes had a significantly lower forced expiratory flow volume in one second, %predicted (%FEV1) compared to other genotypes (Table 2). There were significant differences in the age of bronchial asthma onset among the three groups for rs4950928 and rs1214194 (Tables 1, 2). We next restricted the study to adult-onset bronchial asthma (excluded child-onset asthma) and compared the age of onset. The age of onset of adult bronchial asthma was significantly lower in GG homozygotes of rs4950928 (Table 3) and AA homozygotes of rs1214194 than in the other two genotypes (Table 4).

**Table 1:** Patient characteristics according to the rs4950928 genotype

rs4950928 genotype	CC (n = 270)	CG (n = 90)	GG (n = 21)	*P* value
Age, years	57.6 ± 1.0	58.5 ± 1.7	55.8 ± 3.7	NS
Height, cm	158.4 ± 0.6	159.1 ± 1.0	160.4 ± 2.1	NS
Body weight, kg	59.4 ± 0.7	60.7 ± 1.2	60.8 ± 2.7	NS
Sex, n (%)	110 men (41)	41 men (46)	12 men (57)	NS
Age of onset, years	42.5 ± 1.3	44.3 ± 2.2	31.1 ± 4.5	0.032
Eosinophil count/μL	300.9 ± 17.4	359.5 ± 29.9	277.6 ± 65.1	NS
IgE level, IU/mL	412.1 ± 58.0	435.2 ± 77.9	565.4 ± 233.2	NS
GINA asthma severity, n (%)				
Mild	58 (21.5)	21 (23.3)	6 (28.6)	–
Moderate	105 (38.9)	33 (36.7)	12 (57.1)	–
Severe	107 (39.6)	36 (40.0)	3 (14.3)	–
Pulmonary function				
%FVC	103.3 ± 2.0	104.3 ± 3.4	103.51 ± 9.8	NS
FEV1, L	2.3 ± 0.1	2.3 ± 0.1	2.3 ± 0.2	NS
FEV1/FVC, %	72.9 ± 0.7	70.8 ± 1.2	70.2 ± 2.6	NS
%FEV1	98.9 ± 1.5	98.8 ± 2.6	95.1 ± 5.3	NS
Mild	105.8 ± 3.1	106.8 ± 5.4	91.0 ± 26.5	–
Moderate	100.0 ± 2.3	100.5 ± 4.3	103.3 ± 22.6	–
Severe	94.2 ± 2.3	92.5 ± 4.1	92.2 ± 33.1	–
Equivalent FP CFC dose (μg/day)	382.1 ± 17.0	362.4 ± 29.3	228.0 ± 63.7	NS

Data are expressed as the mean ± standard error

GINA, Global Initiative for Asthma; FVC, forced vital capacity; FEV1, forced expiratory flow volume in one second; NS, not significant among the three groups; FP, fluticasone propionate; CFC, chlorofluorocarbon propellant

**Table 2:** Patient characteristics according to the rs1214194 genotype

rs1214194 genotype	GG (n = 170)	GA (n = 157)	AA (n = 41)	*P* value
Age, years	57.8 ± 1.2	57.7 ± 1.3	58.4 ± 2.7	NS
Height, cm	158.8 ± 0.7	159.0 ± 0.7	157.7 ± 1.5	NS
Body weight, kg	60.5 ± 1.9	59.6 ± 1.0	59.1 ± 1.9	NS
Sex, n (%)	71 men (42)	71 men (45)	15 men (37)	NS
Age of onset, years	43.5 ± 1.6	43.2 ± 1.7	34.2 ± 3.3	0.032
Eosinophil count/μL	315.6 ± 21.7	310.6 ± 23.4	331.7 ± 46.1	NS
IgE level, IU/mL	473.7 ± 97.5	504.4 ± 101.2	235.7 ± 199.2	NS
GINA asthma severity, n (%)				
Mild	35 (20.6)	34 (21.7)	4 (9.8)	–
Moderate	70 (41.2)	61 (38.9)	18 (43.9)	–
Severe	65 (38.2)	62 (39.5)	19 (46.3)	–
Pulmonary function				
%FVC	104.0 ± 2.6	104.0 ± 2.4	97.2 ± 5.9	NS
FEV1, L	2.3 ± 0.1	2.3 ± 0.1	2.1 ± 0.2	NS
FEV1/FVC, %	72.4 ± 0.9	72.2 ± 1.0	70.9 ± 1.8	NS
%FEV1	99.6 ± 2.0	98.8 ± 1.8	87.9 ± 3.6	0.04
Mild	106.7 ± 4.3	102.7 ± 3.8	91.0 ± 11.8	–
Moderate	103.1 ± 3.0	99.3 ± 2.8	89.5 ± 5.6	–
Severe	92.0 ± 3.1	95.1 ± 3.8	85.8 ± 5.4	–
Equivalent FP CFC dose (μg/day)	325.6 ± 20.8	363.1 ± 22.3	432.1 ± 44.1	NS

Data are expressed as the mean ± standard error

GINA, Global Initiative for Asthma; FVC, forced vital capacity; FEV1, forced expiratory flow volume in one second; NS, not significant among the three groups; FP, fluticasone propionate; CFC, chlorofluorocarbon propellant

**Table 3:** Age of adult-onset asthma according to the rs4950928 genotype

rs4950928 genotype	CC (n = 233)	CG (n = 78)	GG (n = 18)	*P* value
Age of onset, years	47.6 ± 1.1	48.4 ± 1.9	35.7 ± 4.0	0.013

Data are expressed as the mean ± standard error

**Table 4:** Age of adult-onset asthma according to the rs1214194 genotype

rs1214194 genotype	GG (n = 148)	GA (n = 135)	AA (n = 34)	*P* value
Age of onset, years	48.2 ± 1.4	48.6 ± 1.5	38.9 ± 2.9	0.009

Data are expressed as the mean ± standard error

### Detection of clinically relevant CHI3L1 levels in BLF supernatants

We performed a bronchoscopy and collected BLF from the two groups to confirm whether GG and AA homozygotes of rs4950928 and rs1214194, respectively, expressed any increased CHI3L1 protein levels. Four patients who were genotyped as CC and 5 patients as GG of rs4950928 harboring wild type of rs1214194 and 6 patients who were genotyped as GG and 4 patients as AA of rs1214194 harboring wild type of rs4950928 were accepted for the BLF studies, which was performed following recommended safety procedures and was well tolerated in all subjects. There were no significant differences in total and differential cell counts between wild type and homozygous. In contrast, CHI3L1 levels in BLF were significantly increased in patients who were homozygous compared to wild type (Table 5).

**Table 5:** Cell differentials and CHI3L1 levels in bronchial lavage fluid

	rs4950928		rs1214194	
	CC (n = 4)	GG (n = 5)	GG (n = 6)	AA (n = 4)
Total cell/mL (×10^4^)	11.5 ± 2.7	10.2 ± 2.4	12.0 ± 2.3	13.3 ± 2.9
Eosinophils, %	1.5 ± 0.5	1.9 ± 0.5	4.0 ± 1.5	6.5 ± 1.9
Macrophages, %	81.3 ± 4.7	75.0 ± 4.2	78.3 ± 4.0	72.5 ± 4.9
Lymphocytes, %	12.5 ± 3.1	15.0 ± 2.8	13.8 ± 3.1	15.0 ± 3.8
Neutrophils, %	2.3 ± 0.9	4.6 ± 0.8	3.3 ± 1.1	5.3 ± 1.4
CHI3L1, pg/mL	140.3 ± 313.5	1144.8 ± 280.4*	179.3 ± 155.30	770.8 ± 190.2^¶^

Data are expressed as the mean ± standard error

* *P *< 0.05 compared with CC, ^¶^*P *< 0.05 compared with GG

## Discussion

In this study, we investigated genetic variants of *CHI3L1* related to clinical characteristics in Japanese asthmatic patients. Several new findings have emerged from this study. First, we demonstrated that asthmatic patients with genetic variants of rs1214194 had a reduced %FEV1. Second, the age of onset of bronchial asthma was significantly younger in homozygotes of rs4950928 and rs1214194 than in other genotypes. When compared with restricted adult-onset asthma, the age of onset was significantly younger in homozygotes than in the wild type and heterozygotes genotypes. Third, CHI3L1 in BLF from *CHI3L1* homozygotes of GG of rs4950928 and AA of rs1214194 increased compared to the wild type in asthmatic patients. Previous population studies of genetic variation in the *CHI3L1* gene and bronchial asthma have shown an association with the promoter SNP rs4950928, intronic SNP rs1214194, and a decreased %FEV1 [15]. However, our studies demonstrated that %FEV1 was decreased in asthmatic patients harboring only homozygotes of rs1214194. One explanation for these differences may depend on medical treatment. Our prospective studies have indicated that *STAT4* TT of rs925847 and *IL13* AA of rs20541 are potential genomic biomarkers that predict lower pulmonary function. High-dose inhaled corticosteroid treatment increased the pulmonary function of patients homozygous for *IL13*AA of rs20541 but not of patients homozygous for *STAT4* TT of rs925847 [22]. Another analysis revealed an association between the homozygous *GLCCI1* rs37972 and rs37973 and the asthmatic treatment steps used with the Japanese population, showing an OR of 2.78 and 2.28, respectively, but not with pulmonary function [23]. We concluded that the recent wide use of anti-IgE and tiotropium produced a clinically meaningful reduction in the exacerbation rate and a sequential improvement in pulmonary function. Thus, decreased FEV1 was dependent on the genetic background and treatment content.

YKL-40/CHI3L1 plays a role in the pathophysiology of bronchial asthma by modulating innate and adaptive immune responses. Surprisingly, to date, the effects of genetic variation in *CHI3L1* on the age of onset of bronchial asthma have not been addressed. To clarify this issue, we compared the ages of adult-onset bronchial asthma between the genotypes for SNPs in *CHI3L1* in Japanese patients. It is generally considered that early-onset asthma included child-onset asthma and adult-onset asthma. However, based on the remaining possibility of the prevention of bronchial asthma development via an improvement of lifestyle habit, it should be distinguished from the perspective of preventive medicine. Genotyping of *CHI3L1* could be considered significant if the development of bronchial asthma can be avoided in men who are found to have a genetic variation of *CHI3L1*, have a family history of bronchial asthma, and are notified early enough to be able to sufficiently adjust their lifestyle habits. Studies of this have added to our understanding of the importance of genetic variations by demonstrating that *CHI3L1* rs4950928 and rs1214194 genotypes play a critical role in early-onset adult asthma. Bronchial asthma should be suspected in anyone with episodic wheezing, shortness of breath, and cough, especially if more than one of the symptoms is worse at night or is precipitated by an upper airway infection; however, these symptoms are relatively nonspecific in adult individuals. Using an examination of SNPs in *CHIL3L1* might promote early detection and intervention in preventing airflow obstructions.

Increased levels of the YKL-40 protein have been found in patients with a broad spectrum of pathologies, including those with rheumatoid arthritis [13], obstructive sleep apnea syndrome [24], solid malignancies [25], atherosclerosis [26], and diabetes mellitus [27, 28]. Previous asthmatic models have demonstrated that diminished antigen-induced responses in chitinase knockout mice were associated with increased eosinophil apoptosis [12]. The potential importance of YKL-40 can also be seen in rheumatoid arthritis, in which elevated serum YKL-40 levels were correlated with the severity of joint involvement [29]. As a result, these observations indicated that YKL-40 might reflect a local inflammation. To our limited knowledge, no other reports have analyzed YKL-40 in BLF, but only in serum [10], sputum [15], and bronchoalveolar lavage fluid (BALF) [30]. With respect to asthma pathophysiology, only BLF reflects chronic airway allergic inflammation and the exact concentration of chemical mediators; therefore, it is still too early to regard the concentration of YKL-40 in the sputum and BALF as a marker of whole bronchial inflammation in bronchial asthma. The aim of the present study was to determine the concentration of YKL-40 in BLF. Further investigations to compare the concentrations in serum, sputum and BLF are needed. The expression of the chitinase breast regression protein (BRP)-39, which is the murine equivalent of YKL-40, was induced by cigarette smoke exposure in a mouse model [31]. The induction by cigarette smoke is IL-1 receptor (R) 1 dependent, which is unique from BRP-39 induction in house dust mite-induced allergic inflammation, which is both IL-1R1 and IL-13 independent. In a human specimen study, YKL-40 promoted bronchial smooth muscle cell proliferation and migration. The cells expressing YKL-40 and BRP-39 in the airways were identified as bronchial epithelial cells and macrophages. Bronchial epithelial expression of YKL-40 is positively correlated with bronchial smooth muscle mass in patients with bronchial asthma [14]. This suggests that cigarette smoke-induced YKL-40 in a Th2 milieu progresses airway remodeling in asthmatic patients. We cannot deny the possibility of advancement in the airway remodeling of bronchial asthmatic patients harboring genetic variants of rs4950928 or rs1214194 and who had an increased YKL-40 in the bronchus due to cigarette smoke. Further examinations are needed to verify the relationships between genetic background, smoking, and declined pulmonary function data.

## Conclusions

In conclusion, this study demonstrated that rs4950928 and rs1214194 homozygotes of *CHI3L1* might be promising genomic biomarkers as predictors of progressing airway remodeling. Before the age of onset of bronchial asthma for patients who have these genetic variants, we need to consider the development of a preventative program that can be implemented before symptoms occur or worse.

## Abbreviations

CHI3L1: chitinase 3-like 1
SNPs: single nucleotide polymorphisms
BLF: bronchial lavage fluid
FVC: forced vital capacity
FEV1: forced expiratory flow volume in one second
NS: not significant
BALF: bronchoalveolar lavage fluid
BRP-39: breast regression protein-39
